# Estimating within-flock transmission rate parameter for H5N2 highly pathogenic avian influenza virus in Minnesota turkey flocks during the 2015 epizootic

**DOI:** 10.1017/S0950268819000633

**Published:** 2019-04-22

**Authors:** A. Ssematimba, S. Malladi, T. J. Hagenaars, P. J. Bonney, J. T. Weaver, K. A. Patyk, E. Spackman, D. A. Halvorson, C. J. Cardona

**Affiliations:** 1 Secure Food Systems Team, College of Veterinary Medicine, University of Minnesota, 1971 Commonwealth Avenue, Saint Paul, MN 55108, USA; 2 Department of Mathematics, Faculty of Science, Gulu University, P.O. Box 166, Gulu, Uganda; 3 Department of Bacteriology and Epidemiology, Wageningen Bioveterinary Research, P.O. Box 65, 8200AB Lelystad, The Netherlands; 4 United States Department of Agriculture, Animal and Plant Health Inspection Service, Veterinary Services, Science, Technology, and Analysis Services, Center for Epidemiology and Animal Health, Natural Resources Research Center, Bldg. B MS-2W4, 2150 Centre Avenue, Fort Collins, CO 80526, USA; 5 Exotic and Emerging Avian Viral Diseases Unit, US National Poultry Research Center, USDA-ARS, 934 College Station Rd. Athens, GA 30605, USA

**Keywords:** Analysis of data, avian flu, mathematical modelling, veterinary epidemiology

## Abstract

Better control of highly pathogenic avian influenza (HPAI) outbreaks requires deeper understanding of within-flock virus transmission dynamics. For such fatal diseases, daily mortality provides a proxy for disease incidence. We used the daily mortality data collected during the 2015 H5N2 HPAI outbreak in Minnesota turkey flocks to estimate the within-flock transmission rate parameter (*β*). The number of birds in Susceptible, Exposed, Infectious and Recovered compartments was inferred from the data and used in a generalised linear mixed model (GLMM) to estimate the parameters. Novel here was the correction of these data for normal mortality before use in the fitting process. We also used mortality threshold to determine HPAI-like mortality to improve the accuracy of estimates from the back-calculation approach. The estimated *β* was 3.2 (95% confidence interval (CI) 2.3–4.3) per day with a basic reproduction number of 12.8 (95% CI 9.2–17.2). Although flock-level estimates varied, the overall estimate was comparable to those from other studies. Sensitivity analyses demonstrated that the estimated *β* was highly sensitive to the bird-level latent period, emphasizing the need for its precise estimation. In all, for fatal poultry diseases, the back-calculation approach provides a computationally efficient means to obtain reasonable transmission parameter estimates from mortality data.

## Background

Highly pathogenic avian influenza (HPAI) virus epizootics often lead to enormous economic losses and, for some of the virus strains with a zoonotic potential, the risk of infecting humans is of an even greater concern. The US 2014–2015 HPAI outbreak started in December 2014 with detections of H5 viruses in captive wild birds and backyard flocks in the northwest, and the first detection in a commercial flock was on 23 January in California [1]. Subsequently, the first HPAI H5N2 virus-infected commercial turkey farm in Minnesota was detected in early March 2015 [1, 2]. A total of 160 commercial turkey operations (104 of which were in Minnesota) were affected, leading to the destruction of 7.4 million turkeys. For the other affected poultry operation types, 43 million table-egg layers and pullets were culled [1]. The impact on the US economy was estimated to be close to US$3.3 billion, of which US$1.6 billion was in direct losses in euthanised animals and the rest was due to restocking costs and lost future production among others [1, 3].

Improved knowledge about within-flock (a flock defined here as a group of birds housed together in a barn) HPAI transmission dynamics would enhance HPAI disease management strategies both before and during an outbreak. Within-flock models of HPAI spread have been used to inform several risk management decisions such as the choice of active surveillance protocol options for early detection (see, e.g. [4]). The implications of improved estimation of parameters for such within-flock models are beneficial to the improved prevention of between-flock transmission [5, 6].

Outbreak detection in an infected flock depends on both the virulence of the pathogen and the rate of virus transmission through the flock [7]. Upon infection, disease dynamics are characterised by, among others, pathogen- and species-specific transmission parameters, which can influence the time to disease detection in the flock and the disease prevalence at that time. These include the transmission rate parameter or adequate contact rate (*β*), defined as the number of new infections caused by an infectious bird per unit time, and the basic reproduction number (*R*_0_), defined as the average number of secondary infections caused by a typical infectious bird during its entire infectious period in a naïve population [8]. Depending on the available data, various methods to estimate these parameters can be used.

The objective of this study was to estimate the within-flock *β* and the corresponding *R*_0_ using daily mortality data obtained from infected turkey flocks from the 2015 H5N2 HPAI virus outbreak in Minnesota, USA. We also assessed how *β* varies between flocks and use the findings from this assessment to obtain the best-fitting distribution for the *β* values to be used in, for example, risk analyses. Transmission rate parameter estimates from this study may be used in HPAI within-flock simulation models to evaluate HPAI control strategies and other decisions related to emergency response and preparedness planning.

## Materials and methods

### Mortality data and model assumptions

Data used in this analysis consisted of date of HPAI detection (hereafter referred to as ‘detection day’) and daily or weekly recorded flock-level mortality from the day of stocking (i.e. placement of birds in a barn) onwards collected from infected turkey farms during epidemiological investigations. Since, in most cases, daily mortality 7 or more days prior to the last recorded day of mortality was indistinguishable from normal mortality, we assumed that HPAI-induced mortality occurred within the last 7 days of the recorded data, hereafter referred to as the ‘period of interest’. For some producers, upon confirmation of flock infection status, efforts shifted towards depopulation preparations and further mortality data collection was not prioritised. Consequently, for those flocks, there were fewer than the required minimum of 3 consecutive days of HPAI-induced mortality and were excluded from further analysis. Daily mortality data were corrected for normal mortality (unrelated to HPAI) throughout the period of interest. Specifically, the mean normal daily mortality was approximated as the average (rounded off to nearest integer) of daily mortality during the third-last week (i.e. week 3 from the last day of recorded mortality) of the records. We chose to use this period since daily mortality immediately after bird-stocking is not a good representation of mortality under normal operating conditions as mortality tends to be higher due to changes in environment among other factors. In cases where the records were insufficient to refer back 3 weeks (e.g. for a flock stocked within the last 2 weeks), average daily mortality was estimated as 0.1% of the total number of birds per day based on normal mortality data from 142 clinically normal tom flocks provided by industry veterinarians for an unrelated analysis [9]. This number was comparable to the 0.07% calculated in this study for flocks where it was possible. The total daily mortality in the period of interest was then adjusted by subtracting the estimated normal mortality for the flock to approximate the HPAI disease mortality.

### Extraction of the desired HPAI-induced mortality chain

Daily mortality in an infected flock may include birds that died due to HPAI disease and those that died due to other causes unrelated to HPAI. Including mortality data from days where most of the mortality is due to normal causes (not disease induced) might reduce the accuracy of the estimated parameters. We defined criteria for selecting flocks and contiguous days with abnormal high mortality during the period of interest so as to minimise the chances of incorrectly fitting transmission parameters to non-disease-induced mortality.

We utilised the concept of a mortality trigger where an abnormal and unexplained increase in daily mortality to levels that exceed predetermined thresholds is used to signal flock infection with HPAI [9–12]. A mortality threshold of 2.5 dead birds per 1000 birds estimated by [9] from predictions of normal mortality among meat turkey tom houses from industry data as one that would result in a low rate of false triggers (<4%) was used. For a flock to be considered for further analysis, its observed mortality had to exceed this threshold at least once during the period of interest.

For the flocks that met these initial criteria, we then extracted the desired mortality chain from the daily mortality data in the period of interest for the back-calculation procedure. For each flock, the Start and End days of the desired HPAI mortality chain were determined as follows. Let the first day on which mortality reaches or exceeds the 0.25% threshold be the Reference Day of the chain. Then, the Start Day of the desired mortality chain was set to be 2 or less days prior to the Reference Day, provided that the daily mortality from the Start Day onwards was above the mean normal mortality (0.1%). In scenarios where the mortality on the days prior to the Reference Day was below 0.1%, the Start Day was taken to be the Reference Day whose mortality is, by definition, ⩾0.25%. These selection criteria were intended to reduce the impact of variations in normal mortality on the estimated *β*. Next, the End Day of the desired mortality we defined to be the last day of recorded mortality of the flock being considered. The End Day was set as Day 0 and the preceding days up to the Start Day were negatively numbered in reference to this Day 0.

Since typical HPAI-induced mortality for this virus strain in turkeys was expected to increase day-by-day, we required that daily mortality is maintained at ⩾0.25% per day on the consecutive days between the Reference Day and the End Day of the mortality chain. Finally, a flock was included in the final analysis only if, upon consideration of these criteria, its extracted mortality chain had 3 or more qualifying days and was confirmed as having been infected during the outbreak. Table 1 presents an example involving four flocks, demonstrating how these procedures were applied to extract the desired mortality chain. In the selected examples, Flock D was disqualified because its mortality went below the threshold on one of the days after the Reference Day (i.e. on Day −1), while Flock E was disqualified because its mortality chain contained fewer than the minimum 3 days required.

**Table 1.** Example demonstrating the application of the flock selection criteria and extraction of mortality chains for back-calculation during the period of interest in four, 10 000 bird flocks with an average normal daily mortality of 0.1% with an HPAI mortality threshold of 0.25%

Day No.^a^	Flock A mortality		Flock B mortality		Flock C mortality		Flock D^b^ mortality	Flock E^b^ mortality
	Recorded	Extracted	Recorded	Extracted	Recorded	Extracted	Recorded	Recorded
−6	5		4		2		10	3
−5	3		2		1		9	11
−4	12^c^	2	9		8		13^c^	2
−3	17	7	13^c^	3	7		29^d^	8
−2	44^d^	34	35^d^	25	31^c^^,^^d^	21	32	4
−1	101	91	121	111	39	29	23	33^c^^,^^d^
0	292	282	403	393	65	55	45	34

a Day 0 refers to the End Day which is also the last recorded data point in the mortality data. The preceding days were negatively numbered in reference to Day 0.

b These flocks do not qualify for back-calculation because they violate the inclusion criteria (see text). Mortality in Flock D drops below the mortality threshold on Day −1. For Flock E, there were only 2 days of mortality data above the mortality threshold of 0.25%.

c Start Day of the mortality chain. It should be 2 or less days prior to the Reference Day and mortality on the Start Day and on the days after must all be greater or equal to the average normal daily mortality 0.1% (10 dead birds per day in this example).

d Reference Day is the first day on which mortality equals or exceeds the set threshold of 0.25% (i.e. 25 dead birds in this example).

### Within-flock dynamics and data analysis

#### Back-calculation procedure

In this procedure, we followed the classical *SEIR* compartmental transmission modelling approaches where, for a given flock on a given day *t*, *S*(*t*) is the number of susceptible birds, *E*(*t*) is the number of latently infected birds, *I*(*t*) is the number of infectious birds and *R*(*t*) is the number of dead birds. Let *N*(*t*) be the total number of birds in the flock and *C*(*t*) be the number of newly infected birds. For the qualifying flocks, their extracted daily mortality data were used to infer the number of birds in the *SEIRC* compartments on each day during the within-flock epidemic. The approach was similar to the method previously described by Bos *et al*. [13].

Upon exposure to infectious material, a bird was assumed to immediately become latently infected for 1 day and subsequently become infectious for 4 days, after which it died. For example, the recorded mortality for Day 0 is for birds that became latently infected at Day −5 and became infectious at Day −4, etc. These bird-level latent and infectious period durations were based on an experimental study in turkeys using the 2015 Minnesota Eurasian/American HPAI H5N2 turkey outbreak field isolate [14] as reported in Cardona *et al*. [15]. Probability distributions were fitted to the experimental data and the best-fitting mean latent and infectious periods were estimated as 1.4 (95% CI 0.9–2.6) and 4.2 (95% CI 3.3–5.5) days, respectively [15]. These were rounded off to 1 and 4 days, respectively, for our analysis since the back-calculation procedure requires whole numbers. These estimates are also supported by the literature review of Spickler *et al*. [7].

All infected birds were assumed to succumb to infection based on the findings of an inoculation experiment using the 2015 Minnesota Eurasian/American HPAI H5N2 turkey outbreak field isolate [14, 15]. This level of fatality has been previously mentioned by Mutinelli *et al*. [16] and De Benedictis *et al*. [17] for field outbreaks. Experimentally, Alexander *et al*. [18] also reported that 100% morbidity and mortality was observed among in-contact turkeys in an experiment using the A/chicken/Pennsylvania/1370/83(H5N2) strain and similar mortality levels were reported for A/turkey/Ontario/7732/66 H5 HPAI virus [19].

Table 2 presents an example of back-calculated data for a sample flock. In the procedure, the selected daily HPAI-induced mortality records (*Dead*) are cumulated to obtain the number of HPAI-dead birds (*total-Dead*) since the Start Day. Based on the set infectious period (4 days), the number of newly infectious birds (*I_new-die*) on a given day is estimated from ‘*Dead*’ 4 days later. Cumulating this number for up to 4 days (i.e. the duration of the infectious period) gives the number ‘*I_total-4-die*’, which is the total number of infectious birds (*I*) on a given day because it was assumed that all infectious birds died.

**Table 2.** An example of back-calculated data based on HPAI-induced mortality data for a selected flock

Day^a^	Dead	total-Dead	I_new-die	I_total-4-die	*I*^b^	*C*^b^	*S*^b^	*N*^b^
−9				0		11		2466
−8			11	11	11	12	2455	2466
−7			12	23	23	13	2443	2466
−6			13	36	36	22	2430	2466
−5			22	58	58	142	2408	2466
−4	11	11	142	189	189		2266	2455
−3	12	23						2443
−2	13	36						2430
−1	22	58						2408
0	142	200						2266

The shaded rows represent the back-calculated estimates of disease states that were used in the analysis.

a In this example, the Start and End days are day −4 and 0, respectively.

b Variables used in the estimation procedure.

Consequently, the number of newly infected birds (*C*) on a given day (*t*) is obtained from ‘*I_new-die*’ a day later. The total number of birds (*N*) on day (*t*) was obtained from the number of birds stocked at the start adjusted for the birds that died. Subtracting the number infected (*I*) from this total gives the number of birds at risk of infection (*S*) on a given day. Mathematica 10.3 (Wolfram Research, Inc., Oxfordshire, United Kingdom) was used to perform the back-calculation procedure on the data.

### Parameter estimation procedure

Within-flock disease dynamics were assumed to follow an ‘*SEIR*’ epidemic model formulation, with a bird as a unit of interest and a frequency-dependent transmission term *β* *SI*/*N* since the flocks were of relatively large size. The birds in the study flocks were housed in groups in a floor system, hence a homogeneous mixing assumption was made. In this model, as explained in [20–22], the number of newly infected birds *C*(*t, Δt*) in a time interval *Δt* = 1 day is approximately binomially distributed with a probability, *p*_inf_(*t*, Δ*t*) = 1 − exp( − *β* *I*(*t*)/*N*(*t*) Δ*t*) and binomial total *S*(*t*): 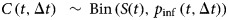. Based on this, the maximum-likelihood value for the transmission rate parameter *β* can be obtained by fitting a GLMM with a complementary log–log link function and log (*I* (*t*) / *N* (*t*)) as an offset variable (e.g. [23]).

Daily records for which the number of infectious birds *I* *>* 0 were compiled into a dataset. The procedure GLIMMIX in SAS 9.4 program (SAS Institute Inc., 2002) (which yielded the same results as the procedure GLMER in statistical software R [24, 25]) was used to carry out the GLMM fitting to estimate *β* and its 95% CI. In the model, the variable ‘flock’ was entered as a random effect. Individual-flock transmission rate parameters were estimated to gain insight into the variation of the predicted parameter, and the within-flock *R*_0_, which was calculated as the product of the estimated transmission rate parameter and the set mean bird-infectious period.

Finally, based on the estimates from the default scenario, we suggest a candidate distribution for *β* to be used in predictive within-flock transmission models and give its 90% prediction interval. The distribution for predicted ln(*β*) was derived by summing two independent normal distributions representing the uncertainty in the model intercept and the random effect for flock (with mean = 0). The distribution for *β* is then obtained by exponentiation and the suggested distribution for *β* is therefore a log-normal distribution.

### Sensitivity analysis and model validation

#### Sensitivity analysis

Four sensitivity analysis scenarios were performed to evaluate alternate model assumptions and the possible impact of outliers. The impact of the assumption that all infected birds succumb to infection was assessed through a scenario in which 43% (as used in Bos *et al*. [13]) of the infected birds survived infection. For assessing the effect of the set latent period, a scenario with a 2-day latent period was compared with the default scenario. A scenario in which daily records with absolute Pearson residuals >10 were omitted from the default scenario dataset was also assessed. Finally, instead of relying on mortality threshold to include flocks in the analysis, we considered a scenario in which the Start Day was 2 days or less prior to the HPAI detection day reported in the original data and only requires that mortality was maintained above average normal daily mortality thereafter.

#### Model validation

For the purposes of validation, the methods used in this study were applied to synthetic datasets generated by simulating both daily normal and HPAI-induced mortality data for 1000 simulated flocks. First, daily normal mortality was modelled using a deterministic normal mortality rate of 0.001 per bird per day over a period of 85 days, which is the average number of mortality records per flock for the flocks included in the final analysis. Then, a 7-day long (i.e. 7 days to represent the period of interest in this study) stochastic ‘*SEIR*’ within-flock epidemic was separately simulated using an individual-based discrete stochastic simulation algorithm [20, 21] with 0.01-day time steps. In this model, the probability that a susceptible bird becomes latently infected in the next time step was *p*_inf_(*t*, Δ*t*) = 1 − exp( − *β* *I*(*t*)/*N*(*t*) Δ*t*) [22]. In the epidemic model simulation, transmission rate parameters *β* = 2.0, 3.0 and 4.0 per day were explored (a set based on this study's outcomes), and the mean latent and infectious periods of 1 and 4 days were maintained. The latent and infectious periods were modelled as being Gamma distributed with shape parameters 1.773 and 7.762 and scale parameters 0.564 and 0.515, respectively [15].

In parameterizing the models, the total number of birds in the simulated flocks was set to 11 805 based on the mean number of birds stocked in the qualifying flocks. All available birds (except one that was assumed infectious) in the flock were assumed to be susceptible at the start of the simulated period.

The predicted daily normal and the predicted HPAI-induced mortality were combined into one simulated dataset by adding the normal and HPAI-induced mortality on the corresponding days. In the validation default approach, which mimics the approach used for outbreak data, the mean normal mortality was subtracted from the overall mortality in synthetic datasets during the period of interest. However, for input *β* = 3.0 per day, we evaluated an alternate scenario in which the synthetic mortality was all assumed to be due to HPAI disease. This alternate assumption was explored to assess the impact of not adjusting for normal mortality. Transmission rate parameters were then estimated by applying the back-calculation and GLMM procedures and compared with the input *β* = 3.0 per day for validation.

## Results

From the available outbreak data, of the 51 HPAI-confirmed turkey flocks with mortality on the last day of the data higher than the mean normal mortality, 29 flocks (i.e. 57%) met the criteria for inclusion in the main analysis (default scenario). The average number of birds stocked in these flocks at the start of the production was 11 547. For each of the scenarios analysed, the following outcomes are reported: the point estimate, its 95% CI and the 5th and 95th percentiles for the predicted individual-flock estimate for *β*, *R*_0_ and its 95% CI, and the standard error and standard deviation estimates for the point estimate of *β* and its variation between flocks.

Table 3 presents the estimated parameters for the different scenarios. In the default scenario, *β* was estimated to be 3.2 with a 95% CI of 2.3–4.3 per day, and a corresponding *R*_0_ of 12.8 (95% CI 9.2–17.2). The 5th and 95th percentiles of the predicted individual flock estimates were, respectively, 1.3 and 10.3 per day. In the sensitivity analyses, increasing the latent period from 1 to 2 days significantly increased *β* to 12.4 (95% CI 6.9–22.3) per day. Assuming that 43% (instead of 0% in the default scenario) of the infected birds survived the infection as well as omitting outliers in the default scenario did not have a significant effect on the estimated parameters. However, omitting outliers improved the model fit as would be expected. Finally, dropping the requirement to exceed the set mortality threshold and only relying on the reported HPAI detection day in the original data yielded a lower but not (statistically) significantly different *β* = 2.1 (95% CI 1.5–2.8) per day and the qualifying number of flocks was similar.

**Table 3.** Estimated transmission rate parameters for the different scenarios: the default scenario assumes a latent period of 1 day, an infectious period of 4 days and all infected birds succumb to the infection

Scenario	Transmission rate parameter *β* (95% CI)	Basic reproduction number *R*_0_ (95% CI)	Std. error for *β* point estimate^a^, ^b^	Std. dev. between flocks^a^, ^b^	5th and 95th percentiles for individual flock *β*^b^	Generalised *χ*^2^/df
Default	3.2 (2.3–4.3)	12.8 (9.2–17.2)	0.142	0.756	1.3–10.3	156
43% surviving infection	3.2 (2.4–4.4)	12.8 (9.6–17.6)	0.143	0.766	1.3–10.5	294
2-day latent period	12.4 (6.9–22.3)	49.6 (27.6–89.2)	0.271	1.405	2.2–95.1	20
Dropping outliers^c^	3.1 (2.2–4.2)	12.4 (8.8–16.8)	0.149	0.790	1.1–10.3	9
Using reported detection day^d^	2.1 (1.5–2.8)	8.4 (6.0–11.2)	0.146	0.761	0.5–6.6	165

a Presented values are on a natural logarithm scale.

b Presented estimates are obtained using procedure GLMER in *R*.

c Estimated from a dataset that omitted daily records whose absolute Pearson residual was >10.

d Setting Start Day 2 or less days prior to reported detection day and mortality staying above normal daily mortality instead of relying on mortality threshold.

From Table 4, we present results of model validation using simulated data. In this case, percentiles were preferred since standard errors are more influenced by sample size. We observed that applying the study methods to simulated data with an input *β* of 3.0 per day and adjusting for normal daily mortality during the period of interest yielded a mean estimate of *β* = 2.9 with its 5th and 95th percentiles as 1.6 and 6.1 per day, respectively, which is consistent with the input *β*. Similarly, for *β* = 2.0 and 4.0 per day, the estimated values were in close agreement with their corresponding inputs. However, in the scenario without adjustment for normal mortality, an input of 3.0 per day resulted in a much lower *β* of 1.6 per day and the input *β* was well outside the estimated 5th and 95th percentiles.

**Table 4.** Estimated parameters from synthetic data obtained from simulations using different values of *β* as inputs and taking mean latent and infectious periods of 1 and 4 days, respectively, while applying the flock selection criteria as used in the default scenario for the outbreak data

Input *β* (per day)	Per cent qualifying flocks^a^	Estimated transmission rate parameter *β* (5th and 95th percentiles for individual flock *β*)^b^	Basic reproduction number *R*_0_ (5th and 95th percentiles for individual flock)	Std. dev. between flocks^c^
3.0	47.2	2.9 (1.6–6.1)	11.6 (6.4–24.4)	0.457
3.0^d^	76.1	1.6 (1.1–2.3)	6.4 (4.4–9.2)	0.228
2.0	1.5	2.2 (1.0–4.8)	8.8 (4.0–19.2)	0.529
4.0	32.8	3.6 (1.8–8.3)	14.4 (7.2–33.2)	0.506

a Percentage of the 1000 synthetic flocks that met the inclusion criteria that is based on HPAI-related mortality in a flock.

b Estimates are obtained using GLMER procedure in *R* and percentiles are preferred since standard error is more influenced by sample size.

c Presented values are on a natural logarithm scale obtained in *R* and standard deviation is preferred since standard error is more influenced by sample size.

d Estimated from synthetic data without adjusting for normal mortality during the period of interest.

## Discussion

Quantifying the transmission characteristics of HPAI in poultry flocks is important for the development of simulation models used to evaluate active surveillance sampling and testing protocol options and other outbreak control measures included in HPAI emergency response plans. Experimental inoculation studies can provide data regarding the timing of infection and/or the onset and duration of shedding which are beneficial for estimating disease state durations (i.e. latent and infectious periods), disease mortality rates, as well as transmission rate parameters (see, e.g. [26]). However, extrapolating experimental transmission dynamics to commercial poultry production flocks is not straightforward due to, for example, environmental (i.e. controlled laboratory *vs.* less or uncontrolled field environment) as well as animal handling differences that may influence disease dynamics. Using mortality data collected from flocks in the field during epidemics provides an option to infer the possible timing of infection for individual birds and estimate the rate of disease transmission for commercial poultry production systems. The use of field data provides an advantage in that it is more representative of actual commercial poultry production management practices, although experimental data are still used as a basis for estimating the length of latent and infectious periods.

This study used daily mortality data obtained from infected turkey farms during the 2015 HPAI H5N2 epizootic in Minnesota, USA, to estimate the transmission rate parameter (*β*).

The mean transmission rate parameter estimated in this study, assuming a latent period of 1 day, and an infectious period of 4 days, was 3.2 (95% CI 2.3–4.3) per day with an *R*_0_ of 12.8 (95% CI 9.2–17.2). We note that dropping potential outliers from the dataset improved the goodness of fit but did not significantly affect *β*. Most importantly, the diagnostic plots of residuals *vs.* fitted value did not show any non-linear patterns, which indicates the link function used is suitable. However, there may be some overdispersion when using our field data, which is not unexpected given the approximations for some of the relevant variables (i.e. number of birds in various disease states) used in the outbreak data-based approaches.

There is a large between-flock variation in the parameter estimates as evidenced in the magnitude of the random effect. We thus recommend that, to improve robustness, distributions that cover the predicted range should be used in risk assessment and surveillance models. Based on the current results (Table 3), and considering the impact of both the uncertainty (measurement error) and between-flock variability, we recommend the use of a log-normal distribution with a mean of 4.36 (i.e. derived from a normal distribution with mean, *μ* = 1.18 and standard deviation, *σ* = 0.756) in simulation modelling studies. The estimated 90% prediction interval of *β* based on this distribution is 0.9–11.5 per day.

When simulated datasets were used for validation, transmission rate parameter estimates were all reproduced within close range of their corresponding input *β*. Slight deviations were likely due to inherent characteristics of the approach, such as the deterministic assumptions made about the length of the latent and infectious periods. Nonetheless, close agreement indicates that the study approach provides a reasonable approximation. This, and the fact that mortality data can be obtained relatively easily, provides support for the use of this approach in the analysis of epizootics associated with similar patterns of rapidly increasing mortality.

Given the impact of HPAI strain characteristics [7] and flock management practices on disease dynamics, this study's outcomes may not necessarily be in agreement with the related studies. However, the estimates from this study are within the range of findings from experimental and field studies involving HPAI in turkeys, especially *β*. For example, in an analysis from an experimental study involving HPAI H7N1 in turkeys that excluded the latent period, Saenz *et al*. [27] estimated a best-fitting *β* of 2.04 (95% CI 1.5–2.7) per day with a mean infectious period of 1.47 (95% CI 1.3–1.7) days, giving an *R*_0_ equal to 3.01 (95% CI 2.2–4.0). Another experimental-based study involving HPAI H7N7 virus in 12-week-old turkeys yielded a mean infectious period of 6.2 days and an estimated *β* of 1.26 (95% CI 0.99–1.59) per day [28]. For field-based studies, using data obtained from the 1999–2000 HPAI H7N1 turkey epidemic in Italy, Bos *et al*. [29] assumed a bird-infectious period of 2 days and estimated a *β* of 1.43 (95% CI 1.17–1.74) per day. In a study based on epidemic data for 2003 HPAI H7N7 outbreak in the Netherlands using a 4-day infectious period, the overall *β* for turkeys was estimated as 3.37 (95% CI 0.97–11.74) per day [13]. There are some important sources of variability that could explain the range of estimates between studies, such as differences in experimental design, the difference in HPAI virus strain characteristics (i.e. the duration of the latent and infectious periods) and regional differences in flock management practices.

We have some methodological improvements relative to the previous studies [13, 29, 30]. For example, in this study, we adjusted for non-HPAI-related mortality during the period of interest. Although, we cannot precisely determine the fraction of observed mortality that is due to HPAI disease, analysis on synthetic data indicates that adjusting for normal mortality based on mean values yields more accurate parameter estimates. Additionally, in order to ensure that the back-calculation step is only applied to mortality that is mainly due to HPAI disease, we applied the absolute mortality threshold criteria. These criteria were aimed at minimizing the impact of variability in normal mortality on the estimated transmission rate parameter and, most importantly, excluding days whose recorded daily mortality is likely to have occurred independent of HPAI disease.

In our validation analysis using simulated data, not correcting for daily normal mortality during the period of interest lowers the predicted transmission rate parameter estimates, yielding a *β* of 1.6 per day from an input of 3.0 per day (Table 4). Therefore, assuming that the mortality observed during the period of interest is fully due to HPAI does not seem to be justified, and the question of whether or not to adjust for normal daily mortality deserves careful consideration in the future analyses where field outbreak data are used to estimate the transmission rate parameter *β*. Finally, the alternative scenario of setting the Start Day as 2 or less days prior to the reported day of detection yielded a statistically insignificantly lower *β*. However, based on actual field practices and on the outcomes of this validation step, we recommend that surveillance protocols also consider the use of mortality triggers as a supplementary detection mechanism to initiate further diagnostic investigations for HPAI [9–12].

The overall raw mortality dataset included mortality data from some barns on infected premises which did not demonstrate the typical exponential rise in HPAI mortality above the normal mortality threshold. We hypothesise that the absence of such a trend in those flocks may have been due to early detection that occurred because of active surveillance via testing dead birds. This active surveillance was implemented for flocks in an HPAI control area to promote early detection as well as to meet the requirements for continuity of business guidelines. Additionally, for the final set of flocks included in the back-calculation procedure, we verified that the barns were infected with HPAI H5N2 virus (RRT-PCR test positive). The qualifying number of flocks being relatively low was also, in part, a consequence of incomplete data whereby some flocks lacked test results and/or the total number of birds stocked.

In the sensitivity analysis, the study outcomes were robust to the assumption on the fraction surviving the infection, but were sensitive to the set latent period. Also, changing the infectious period to either 3 or 5 days did not significantly affect the estimated *β* (results not shown). Insensitivity to the surviving fraction may indicate that if only a fraction of infected birds die, correcting for this affects both the infection pressure and the number of newly infected birds in similar ways, thus having only a small effect on the estimate. Sensitivity to the duration of the latent period was also found in other related studies (e.g. [13]). Note that the duration of the latency period is both pathogen- and species-specific. Therefore, it is important to use relevant estimates for the HPAI strain and bird species.

Methodological limitations of this and other related studies that use back-calculation with GLM-based estimation include the assumption of deterministic latent and infectious periods, and disregarding important sources of between-bird variability (e.g. HPAI susceptibility and infectivity). Moreover, using whole integers for these durations represents a discrete approximation of a continuous process, where the exact timing of death is not considered. Other than improving the resolution of the timing of death by increasing the frequency of collecting mortality data (which is impractical), the other limitations may require different and possibly computationally intensive approaches. We also assumed a frequency-dependent transmission model, implicitly equivalent to assuming that contact rates are limited by behavioural or social factors and do not depend significantly on population density. Besides, if the population size remains more or less constant as an epidemic passes through, then density- and frequency-dependent models are equivalent [22, 31–33]. Nonetheless, the study approaches performed reasonably well in validation using synthetic data (Table 4). In other words, there is enough evidence to support the conclusion that the methods and assumptions upon which this study was based are reasonable approximations. It is also important to note that among the closely related studies, this validation step on simulated data is unique to this study.

We conclude that back-calculation is a computationally efficient method that uses accepted GLM-based procedures to produce reasonable estimates for the within-flock transmission rate parameter *β* for HPAI in turkeys and possibly other livestock diseases that are characterised by rapidly increasing mortality. The within-flock estimates for the transmission rate parameter and the basic reproduction number from this study can be used to inform a number of analyses for decision support. Examples include between-flock disease transmission models (e.g. InterSpread Plus) used to evaluate the effectiveness of disease control strategies on geographic spread, and within-flock simulation models that are used to predict time to detection of HPAI virus in infected turkey flocks to evaluate different surveillance methods. In turn, outcomes from those studies can be used to inform decisions on the selection of appropriate mitigation measures, which could limit the geographical spread of the disease. Finally, there is a need for further research to gain more insight into the factors underlying the observed wide variation in the estimated transmission rate parameter among poultry flocks.
